# Associations of HIV and iron status with gut microbiota composition, gut inflammation and gut integrity in South African school‐age children: a two‐way factorial case–control study

**DOI:** 10.1111/jhn.13171

**Published:** 2023-04-16

**Authors:** Charlene Goosen, Sebastian Proost, Jeannine Baumgartner, Kashish Mallick, Raul Y. Tito, Shaun L. Barnabas, Mark F. Cotton, Michael B. Zimmermann, Jeroen Raes, Renée Blaauw

**Affiliations:** ^1^ Division of Human Nutrition, Department of Global Health, Faculty of Medicine and Health Sciences Stellenbosch University Cape Town South Africa; ^2^ Laboratory of Molecular Bacteriology, Department of Microbiology and Immunology Rega Institute, KU Leuven Leuven Belgium; ^3^ Center for Microbiology, VIB Leuven Belgium; ^4^ Laboratory of Human Nutrition, Department of Health Sciences and Technology ETH Zurich Zurich Switzerland; ^5^ Department of Nutritional Sciences King's College London London UK; ^6^ Department of Paediatrics and Child Health, Family Centre for Research with Ubuntu Stellenbosch University Cape Town South Africa

**Keywords:** gut microbiota, HIV, inflammation, iron deficiency, school‐age children, South Africa

## Abstract

**Background:**

Human immunodeficiency virus (HIV) and iron deficiency (ID) affect many African children. Both HIV and iron status interact with gut microbiota composition and related biomarkers. The study's aim was to determine the associations of HIV and iron status with gut microbiota composition, gut inflammation and gut integrity in South African school‐age children.

**Methods:**

In this two‐way factorial case–control study, 8‐ to 13‐year‐old children were enrolled into four groups based on their HIV and iron status: (1) With HIV (HIV+) and ID (*n* = 43), (2) HIV+ and iron‐sufficient nonanaemic (*n* = 41), (3) without HIV (HIV−) and ID (*n* = 44) and (4) HIV− and iron‐sufficient nonanaemic (*n* = 38). HIV+ children were virally suppressed (<50 HIV RNA copies/ml) on antiretroviral therapy (ART). Microbial composition of faecal samples (16S rRNA sequencing) and markers of gut inflammation (faecal calprotectin) and gut integrity (plasma intestinal fatty acid–binding protein [I‐FABP]) were assessed.

**Results:**

Faecal calprotectin was higher in ID versus iron‐sufficient nonanaemic children (*p* = 0.007). I‐FABP did not significantly differ by HIV or iron status. ART‐treated HIV (redundancy analysis [RDA] *R* ^2^ = 0.009, *p* = 0.029) and age (RDA *R* ^2^ = 0.013 *p* = 0.004) explained the variance in the gut microbiota across the four groups. Probabilistic models showed that the relative abundance of the butyrate‐producing genera *Anaerostipes* and *Anaerotruncus* was lower in ID versus iron‐sufficient children. *Fusicatenibacter* was lower in HIV+ and in ID children versus their respective counterparts. The prevalence of the inflammation‐associated genus *Megamonas* was 42% higher in children with both HIV and ID versus HIV− and iron‐sufficient nonanaemic counterparts.

**Conclusions:**

In our sample of 8‐ to 13‐year‐old virally suppressed HIV+ and HIV− children with or without ID, ID was associated with increased gut inflammation and changes in the relative abundance of specific microbiota. Moreover, in HIV+ children, ID had a cumulative effect that further shifted the gut microbiota to an unfavourable composition.

## Key points

1. Iron deficiency (ID) was associated with higher gut inflammation measured by faecal calprotectin.
2. Gut mucosal integrity measured by intestinal fatty acid–binding protein did not differ by human immunodeficiency virus (HIV) or iron status.
3. In HIV+ children, ID had a cumulative effect that promoted an unfavourable gut microbiota composition.

**Abbreviations**
3TC: lamivudine; ABC abacavir
AGP: α‐1‐acid glycoprotein
ANCOVA: analysis of covariance
ART: antiretroviral therapy
ASV: Amplicon Sequence Variants
ATV/r: atazanavir boosted with ritonavir
AZT: zidovudine
BRINDA: Biomarkers Reflecting Inflammation and Nutritional Determinants of Anaemia
CRP: C‐reactive protein
EFV: efavirenz
FTC: emtricitabine
Hb: haemoglobin
HDI: highest density intervals
HIV: human immunodeficiency virus
HIV−: living without HIV
HIV+: living with HIV
ID: iron deficiency
I‐FABP: intestinal fatty acid–binding protein
IQR: interquartile range
LPV/r: lopinavir boosted with ritonavir
NNRTI: non‐nucleoside reverse‐transcriptase inhibitor
NRTI: nucleoside reverse‐transcriptase inhibitors
NVP: nevirapine
PCoA: principal coordinate analysis
PF: plasma ferritin
PI/r: ritonavir‐boosted protease inhibitor
RDA: redundancy analysis
SD: standard deviation
sTfR: soluble transferrin receptor
TDF: tenofovir

## INTRODUCTION

Globally, ~37.7 million people are living with human immunodeficiency virus (HIV+), and two‐thirds of this population are from sub‐Saharan Africa. The number of HIV+ children <15 years is estimated at 1.7 million, of whom 310,000 (18%) are from South Africa.^1^ In many African countries, an additional challenge is malnutrition, with 52% of African children estimated to be iron deficient.^2^ Because HIV is associated with chronic systemic inflammation,^3^, ^4^, ^5^ HIV+ children are more vulnerable to iron deficiency (ID) than children without HIV (HIV−). Systemic inflammation can down‐regulate iron absorption, adversely affect iron status and cause anaemia of inflammation.^6^ This is especially detrimental in resource‐poor settings where iron intake from plant‐based diets is low and poorly bioavailable, which in turn causes nutritional anaemia.^7^

The interplay between colonic iron availability and compositional changes in the gut microbiota has become a topic of interest, though more frequently discussed in the context of an iron surplus. However, in vitro colonic fermentation and animal studies have found that very low colonic iron conditions significantly alter gut bacteria composition and function, especially butyrate producers and short‐chain fatty acid metabolism.^8^, ^9^ A potential explanation is that enzymes in the butyrate production pathway are iron dependent.^10^ It is unclear whether these findings are applicable to ID children, where complex human–microbiota interactions and fine regulation of iron absorption come into play.

The gut is also the compartment most persistently affected by HIV in the long term, despite viral suppression. Soon after HIV infection, the gut‐associated lymphoid tissue is severely depleted of CD4+ T cells, including T17 helper cells that are essential to maintain the gut mucosal barrier.^11^ The early loss of CD4+ T cells marks the beginning of HIV‐associated enteropathy.^12^ Although antiretroviral therapy (ART) suppresses HIV replication and reverses CD4+ T‐cell depletion in the peripheral blood compartment, its kinetics are slower in the gut mucosa. This leads to unsuccessful or partial replenishment of the gut environment. Consequences of HIV‐associated enteropathy include gut inflammation and increased intestinal permeability, despite ART.^13^ A loss of gut mucosal integrity could facilitate microbial translocation, with chronic immune activation, poorer restoration of CD4+ T cells and disturbances of the host–microbiota homeostasis as potential consequences.^14^, ^15^, ^16^

Studies suggest that ART alters gut microbiota composition to a bacterial community structure distinct from HIV−, as well as ART‐naive HIV+ reference groups.^16^, ^17^ In HIV+ populations, gut microbiota composition appears to shift towards higher proportions of proinflammatory and lower proportions of anti‐inflammatory bacterial species.^16^, ^18^, ^19^ This state of dysbiosis promotes pathogenic and inflammatory pathways.^20^, ^21^

Current and future paediatric HIV populations will increasingly receive ART from early infancy. Thus, understanding the effects of prolonged ART exposure is becoming more important. Both HIV and iron status interact with gut inflammation, gut integrity and microbiota composition, yet to our knowledge, associations between iron status and gut health in the context of HIV have not been studied. Considering the high burden of HIV and ID in sub‐Saharan Africa, it is important to examine the relationship of both HIV and iron status with gut health, as well as the potential interactions between HIV and iron status. Therefore, the aim of this study was to determine the associations of HIV and iron status with gut microbiota composition, gut inflammation and gut integrity in South African school‐age children.

## METHODS

### Study design and participants

This two‐way factorial case–control study was performed within a cohort of 8‐ to 13‐year‐old HIV+ and HIV− children at the Family Centre for Research with Ubuntu in Cape Town, South Africa.^22^ Children were eligible if they reported no recent acute illness, if they reported no iron supplementation use in the past 3 months and, in the HIV+ children, if they were virally suppressed (<50 HIV RNA copies/ml plasma) based on routine annual monitoring data obtained from the National Health Laboratory Service electronic portal. In HIV− children, the absence of HIV was confirmed using a rapid HIV assay (First Response HIV Card 1–2.0, Premier Medical Corporation Pvt Ltd). Children were excluded if severely underweight or obese (body‐mass‐index‐for‐age *Z*‐score <−3 or >2)^23^ and severely anaemic (haemoglobin [Hb] <80 g/L).^7^ Screening measurements included serum ferritin and Hb, and based on their HIV and iron status, 180 children were enrolled in the following four groups: (1) HIV+ and ID (*n* = 45), (2) HIV+ and iron‐sufficient nonanaemic (*n* = 45), (3) HIV− and ID (*n* = 45) and (4) HIV− and iron‐sufficient nonanaemic (*n* = 45). For enrolment purposes, ID was defined as inflammation‐unadjusted ferritin ≤40 µg/L, iron sufficiency as unadjusted ferritin >40 µg/L and the absence of anaemia as Hb ≥115 g/L.

For this present study, further exclusion criteria included (1) detectable HIV viral load, (2) antibiotic use in the 4 weeks prior to faecal sample collection, (3) probiotic use 1 week prior to faecal sample collection, (4) vegetarianism or veganism and (5) self‐reported gastrointestinal disorders. HIV viral load was measured (Roche COBAS AmpliPrep/TaqMan HIV‐1 Test, v2, Hoffmann‐La Roche, Basel, Switzerland), and six children were excluded because of viral loads ≥50 copies/ml. Three children were excluded because of antibiotic use. Of the remaining 171 children, 5 children provided an insufficient faecal sample for the necessary measurements, resulting in a final subsample of 166 children. The number of participants per group was (1) HIV+ and ID (*n* = 43), (2) HIV+ and iron‐sufficient nonanaemic (*n* = 41), (3) HIV− and ID (*n* = 44) and (4) HIV− and iron‐sufficient nonanaemic (*n* = 38). Based on the two‐way factorial design and assuming 80% power and a type I error rate of 5%, the final sample size allowed us to detect an effect size of 0.3 between groups.

Participant, socio‐demographic, anthropometric, dietary intake, anaemia, iron status and systemic inflammation indicators were collected with the using detailed methods previously described.^22^ In brief, socio‐demographic and HIV information was obtained using a structured questionnaire, and weight and height were measured using a Micro 1023 electronic platform scale and stadiometer (Scalerite) and standardised techniques.^24^ Habitual dietary intake information was collected using an abbreviated quantified food frequency questionnaire. The questionnaire was developed for the specific study population using a methodical multiphase approach with the using details previously described.^25^ Hb concentrations were measured in whole blood using a Siemens Advia 2120i Haematology System (Siemens), and plasma ferritin (PF), soluble transferrin receptor (sTfR), C‐reactive protein (CRP) and α‐1‐acid glycoprotein (AGP) were measured using a multiplex immunoassay.^26^ PF values were adjusted for inflammation using the Biomarkers Reflecting Inflammation and Nutritional Determinants of Anaemia (BRINDA) correction approach.^27^ This approach uses linear regression to adjust PF using the CRP and AGP concentrations. Intestinal fatty acid–binding protein (I‐FABP) was measured using an ELISA (enzyme‐linked immunosorbent assay) immunoassay (Hycult Biotech).

### Faecal sample collection and analyses

Faecal samples were collected at home the night or morning before the study visit. The children received a lined plastic container with a lid that sealed airtight and an OxoidTM AnaeroGenTM 2.5‐L Sachet (ThermoFisher Scientific Inc.) to generate an anaerobic environment in the container after sample deposit. A cooler bag, icepacks and illustrated instructions in their home language were provided in addition. On the day of the visit, stool samples were aliquoted and frozen at −70°C for the calprotectin and gut microbiota analyses. Faecal calprotectin was measured using an ELISA immunoassay (Eurospital). Elevated gut inflammation was classified as faecal calprotectin >200 μg/g.^28^

### DNA extraction, library preparations and sequencing

DNA extraction was performed using the MagAttract PowerMicrobiome DNA/RNA Kit (Qiagen). For microbiota analysis, the V4 region of the 16S rRNA gene was amplified with the primer pairs 515F and 806R (GTGYCAGCMGCCGCGGTAA and GGACTACNVGGGTWTCTAAT, respectively), modified to contain a barcode sequence between each primer and the Illumina adaptor sequences to produce dual‐barcoded libraries. Followed by size selection using Agencourt AMPure to remove fragments below 200 bases, 16S rRNA sequencing was performed on an Illumina MiSeq platform (MiSeq Reagent Kit v2, 500 cycles, 15.38% PhiX, 2 × 250 PE) at the VIB Nucleomics core laboratory (Leuven, Belgium). De‐multiplexing was performed using LotuS. This was followed by quality inspection; the removal of chimeras, primers and the first 10 bases following the primer; and the merging of paired sequences using DADA2 (v1.6). The resulting sequences were further grouped into Amplicon Sequence Variants (ASV).^29^ Finally, taxonomy was assigned to all ASVs (using Ribosomal Database Project's trainset 16) and agglomerated to genus level.

### Statistical analysis

#### Participant characteristics and gut health markers

Statistical analyses were performed using IBM SPSS Statistic software, version 27 (IBM Corp.). Normally distributed continuous variables were described using means and standard deviations (SD), non‐normally distributed variables with medians and interquartile ranges and categorical variables with frequencies and percentages. Non‐normally distributed outcome variables were log‐transformed prior to analysis. The characteristics of the four groups were compared using two‐way factorial analysis of variance for continuous variables and two‐way binary logistic regression for categorical variables. The associations of HIV and iron status with gut health markers were assessed using two‐way factorial analysis of covariance (ANCOVA) for continuous outcome variables and two‐way binary logistic regression for categorical outcome variables, adjusting for age, sex, ethnicity and deworming. If the ANCOVA or logistic regression did not show a significant interaction effect, it was repeated without the interaction factor. In the case of a significant interaction effect (observed only for a categorical outcome variable in this study), between‐group differences were analysed using a χ^2^ test with Bonferroni adjustment for multiple comparisons. Statistical significance was set at *p* < 0.05.

#### Gut microbiota

Statistical analyses were performed using R statistical software (http://www.r-project.org/). Genera with low prevalence (detected in less than 20% of the samples) were excluded from the analysis. The α‐diversity for each sample was calculated using the Shannon diversity index upon the rarefied abundances. A principal coordinate analysis (PCoA), using the Bray–Curtis distance, was carried out on 16S rRNA gene abundances after aggregating counts at the genus level (using DADA2). The Kruskal–Wallis test with post hoc Dunn's test (with fdr_bh to correct for multiple testing) was used to test median differences of α‐ and β‐diversity between groups. Enterotypes were obtained by combining 16S rRNA gene data from this sample with data from the Flemish Gut Flora Project^30^ and applying an approach based on Dirichlet multinomial mixtures.^31^

Redundancy analysis (RDA) was used to identify variables in the metadata that explained the variance in gut microbiota composition between the four groups. The independent effect size of significant variables on microbial composition was determined using the function *capscale* (using Euclidean distance on centred log‐ratio‐transformed abundance data), part of the *vegan* package, whereas the nonredundant effect was obtained combining the *rda* and *ordiR2step* from the same package. Statistical significance was set at a false discovery rate <0.1. To force a feature to be considered before others, this procedure is split into two steps: first, *ordiR2step* is run with a null model (without features) to a model with features that need to be accounted first (HIV status in this study – see ‘Results’ section). Next, *ordiR2step* is run again starting from the model with the forced features to the model with all features. Finally, the output from both runs is merged.

To assess the associations of HIV and iron status with various genera, a probabilistic model was used implemented in Python (version 3.10.5) with PyMC (version 4.0.0).^32^ Here five models, based on negative binomial distributions, with various degrees of complexity, were used. The simplest model contains a single feature (*p*_base_) to model the number of reads found from a given genus in all samples which is used with a binomial likelihood. For more complex models, additional features (*m*_hiv_, *m*_id_ and *m*_interaction_) that allow HIV and iron status to affect the binomial likelihood's probability *p* were included.

Given HIV and iron status are encoded as 0 or 1 (for absent and present, respectively), the models’ probabilities were defined as follows:

- *p* = *p*_base_
- *p* = *p*_base_ + *m*_hiv_ × HIV status
- *p* = *p*_base_ + *m*_id_ × iron status
- *p* = *p*_base_ + *m*_hiv_ × HIV status + *m*_id_ × iron status
- *p* = *p*_base_ + *m*_hiv_ × HIV status + *m*_id_ × iron status + *m*_interaction_ × (HIV status × iron status)

All five models were run on all genera using PyMC's No‐U‐Turn sampler^33^ with 4000 samples, 2000 tuning steps and 4 chains. Noninformative priors were used for all features. For each genus, the simplest model with the best fit was selected by visual inspection of the model performance using the function *plot_compare* from ArViz (version 0.12.1).^34^ Genera where either HIV or iron status was retained as a component of the model were considered for further analysis. Probability density functions and highest density intervals (HDI) for the models inspected in detail were generated using *plot_trace* and *summary* from the ArViz package.

To assess if a difference in the prevalence of participants with *Megamonas* better explained our observations for that genus than differences in abundance between participant groups, another model was used. In this model each of the four groups was assigned two weights *w* for participants with and without *Megamonas* using a Dirichlet distribution with a flat prior. Two possible binomial distributions, one with the probability of success set to zero (for participants lacking the genus) and the other with the success probability set to a variable *p*_base_ (from a HalfNormal distribution with sigma 0.001), were combined with the weights *w* for each group and compared with the data using a mixture likelihood. Deterministic variables were set to extract the difference in prevalence of participants with *Megamonas* between HIV− and iron‐sufficient nonanaemic participants and participants from the other groups. Sampling was performed using the same settings as the previous analysis except for the parameter *target_accept* which was increased to 0.9.

All figures for this study were generated using Python 3.10.5 with Seaborn 0.11.2 and Matplotlib 3.5.1. Statsannotations 0.4.4 was used to include results from statistical tests in box plots.

## RESULTS

### Participant characteristics

Participant characteristics are summarised in Table 1. Compared with HIV− children, HIV+ children were from smaller households (*p* = 0.040) had lower height‐for‐age *Z*‐scores (*p* < 0.001), and higher sTfR levels (*p* = 0.001), CRP (*p* = 0.007) and AGP concentrations (*p* = 0.031). HIV+ children reported lower intake of animal protein (*p* < 0.001) and haem iron (*p* = 0.002) compared with HIV− children. Compared with iron‐sufficient nonanaemic children, more ID children received care from a single primary caregiver (*p* = 0.028). isiXhosa African children comprised 43% of the study population, and 57% of the children were of Capetonian mixed ancestry. The proportions of these two ethnic groups differed significantly between groups, with fewer isiXhosa African children in the HIV− iron‐sufficient nonanaemic group compared with the other three groups. Of all children, 96% had been dewormed in the past 6 months. In the HIV+ children, ritonavir‐boosted protease‐inhibitor‐based ART regimens were more common (71%) than non‐nucleoside reverse‐transcriptase inhibitor‐based regimens (29%).

**Table 1:** Characteristics of the four groups of South African children enrolled based on HIV and iron status.

	HIV+ and ID	HIV+ and iron‐sufficient nonanaemic	HIV− and ID	HIV− and iron‐sufficient nonanaemic		*p*‐Values*	HIV × iron status
	*n* = 43	*n* = 41	*n* = 44	*n* = 38	HIV	Iron status	
*Participant information*							
Age (y), median (IQR)	11.6 (9.8–12.5)	11.4 (10.8–12.4)	11.2 (9.7–12.3)	10.6 (9.5–12.2)	0.06	0.94	0.23
Male/female, *n* (%)	20 (47)/23 (54)	27 (66)/14 (34)	21 (48)/23 (52)	20 (53)/18 (47)	0.46	0.12	0.34
isiXhosa African/Capetonian mixed ancestry, *n* (%)†	18 (42)/25 (58)_a_	27 (66)/14 (34)_a_	23 (52)/21 (48)_a_	3 (8)/35 (92)_b_	0.33	0.029	<0.001
Dewormed in the past 6 months, *n* (%)	40 (93)	40 (98)	43 (98)	37 (97)	0.42	0.47	0.50
HIV RNA (copies/ml)	<50	<50	–	–	–	–	–
Age at antiretroviral therapy start (y), median (IQR)	1.0 (0.0–2.5)	1.0 (0.0–1.0)	–	–	–	0.94	–
NNRTI‐based/PI/r‐based, *n* (%)‡	14 (33)/29 (67)	10 (24)/30 (73)	–	–	–	0.45	–
*Household information*							
Formal/informal housing, *n* (%)§	26 (61)/17 (40)	23 (56)/18 (44)	30 (68)/14 (32)	26 (68)/12 (32)	0.19	0.78	0.77
Number of household members, median (IQR)	5 (4–6)	5 (4–6)	6 (4–7)	6 (5–7)	0.040	0.47	1.00
Primary caregiver single/in partnership, *n* (%)	24 (56)/19 (44)	16 (39)/25 (61)	19 (43)/25 (57)	10 (26)/28 (74)	0.09	0.028	0.91
Breadwinner unemployed, *n* (%)	20 (47)	17 (42)	22 (50)	10 (26)	0.48	0.06	0.20
*Anthropometry*							
Height‐for‐age *Z*‐score, mean ± SD	−1.4 ± 1.0	−1.1 ± 0.9	−0.7 ± 1.0	−0.4 ± 0.9	<0.001	0.12	0.98
Body‐mass‐index‐for‐age *Z*‐score, mean ± SD	−0.4 ± 1.0	−0.4 ± 1.0	−0.2 ± 1.2	−0.1 ± 1.0	0.12	0.70	0.57
*Anaemia and iron status*							
Haemoglobin (g/L), mean ± SD	119 ± 11	126 ± 8	121 ± 8	124 ± 8	0.74	<0.001	0.23
Plasma ferritin (adjusted for inflammation) (μg/L),‖ median (IQR)	17 (13–27)	38 (27–66)	20 (17–26)	35 (27–49)	0.88	<0.001	0.07
Soluble transferrin receptor (mg/L), median (IQR)	7.0 (6.0–8.9)	7.0 (5.4–8.7)	6.5 (5.8–7.2)	6.1 (5.3–6.9)	0.001	0.23	0.41
*Systemic inflammation*							
C‐reactive protein (mg/L), median (IQR)	0.12 (0.02–1.14)	0.04 (0.03–1.04)	0.04 (0.02–0.35)	0.04 (0.02–0.43)	0.007	0.73	0.89
α‐1‐acid glycoprotein (g/L), median (IQR)	0.6 (0.5–0.8)	0.6 (0.4–0.9)	0.5 (0.4–0.8)	0.5 (0.4–0.7)	0.031	0.78	0.47
*Selected daily nutrient intake*							
Total protein (g), median (IQR)	69 (53–76)	62 (52–76)	80 (62–98)	81 (66–102)	0.003	0.73	0.47
Animal protein (g), median (IQR)	32 (22–39)	27 (21–37)	40 (26–51)	44 (34–64)	<0.001	0.55	0.15
Plant protein (g), median (IQR)	34 (29–45)	35 (25–42)	36 (30–45)	34 (22–41)	0.69	0.07	0.69
Total iron (mg), median (IQR)	16 (14–21)	17 (14–21)	18 (15–21)	17 (13–22)	0.84	0.19	0.92
Haem iron (mg), median (IQR)	2.3 (1.6–4.0)	2.0 (1.5–3.4)	3.3 (2.0–4.3)	3.2 (2.5–5.1)	0.002	1.00	0.19
Nonhaem iron (mg), median (IQR)	13 (12–18)	14 (11–17)	15 (12–17)	12 (9–18)	0.44	0.13	0.73
Total fibre (g) median (IQR)	25 (20–31)	25 (19–30)	28 (22–36)	25 (18–36)	0.32	0.12	0.90

Abbreviations: HIV, human immunodeficiency virus; ID, iron deficient; IQR, interquartile range; NNRTI, non‐nucleoside reverse‐transcriptase inhibitor; PI/r, ritonavir‐boosted protease inhibitor; SD, standard deviation.

* Non‐normally distributed outcome variables were log‐transformed prior to analysis. Associations of the factors HIV and iron status with household and socio‐demographic characteristics were assessed using two‐way analysis of variance for continuous variables and two‐way logistic regression analysis for categorical variables.

^†^ Between‐group differences were analysed using a χ^2^ test with Bonferroni adjustment for multiple comparisons. Values in a row without a common letter (a, b) differ significantly (*p* < 0.05).

^‡^ HIV+ and iron‐sufficient nonanaemic (*n* = 40), incomplete information. All regimens included two nucleoside reverse‐transcriptase inhibitors (NRTIs) in combination with either a non‐nucleoside reverse‐transcriptase inhibitor (NNRTI‐based) or a ritonavir‐boosted protease inhibitor (PI/r‐based). NRTIs included abacavir (ABC), lamivudine (3TC), zidovudine (AZT), tenofovir (TDF) and emtricitabine (FTC); NNRTIs included efavirenz (EFV) and nevirapine (NVP); and PIs included lopinavir boosted with ritonavir (LPV/r) and atazanavir boosted with ritonavir (ATV/r).

^§^ Formal housing represents a brick house, whereas informal housing represents a Wendy house or dwelling built with scrap building material and typically not equipped with water and/or electricity.

^‖^ Plasma ferritin adjusted for inflammation using the Biomarkers Reflecting Inflammation and Nutritional Determinants of Anaemia correction approach.^27^

### Gut inflammation and gut integrity

Table 2 presents the measured gut health‐related biomarkers. Faecal calprotectin was significantly higher in ID children than in iron‐sufficient nonanaemic children (*p* = 0.007). I‐FABP did not significantly differ by HIV or iron status.

**Table 2:** Gut inflammation and gut integrity.

	HIV+ and ID	HIV+ and iron‐sufficient nonanaemic	HIV− and ID	HIV− and iron‐sufficient nonanaemic	*p*‐Values^a^		
	*n* = 43	*n* = 41	*n* = 44	*n* = 38	HIV	Iron status	HIV × iron status
Faecal calprotectin (µg/g) median (IQR)^b^	22 (7–57)	6 (3–18)	20 (3–75)	5 (1–50)	0.85	0.006	0.77
50–200 µg/g, *n* (%)	7 (17)	4 (10)	8 (18)	7 (18)	0.36	1.00	0.36
>200 µg/g, *n* (%)	4 (10)	4 (10)	5 (11)	2 (5)	0.99	0.50	0.45
Intestinal fatty acid–binding protein (pg/ml), median (IQR)	859 (482–1164)	799 (366–1110)	633 (457–1010)	935 (603–1219)	0.47	0.44	0.09

Abbreviations: HIV, human immunodeficiency virus; ID, iron deficient; IQR, interquartile range.

^a^ Non‐normally distributed outcome variables were log‐transformed prior to analysis. Associations of the factors HIV and iron status with gut health markers were assessed using two‐way analysis of covariance for continuous variables and two‐way logistic regression analysis for categorical variables, adjusting for age, sex, ethnicity and deworming.

^b^ Total study population *n* = 162, HIV+ and ID *n* = 42, HIV+ and iron‐sufficient nonanaemic *n* = 39 and HIV− and ID *n* = 43, because insufficient stool sample sizes were provided in four cases.

### Gut microbiota composition

**Figure 1:**
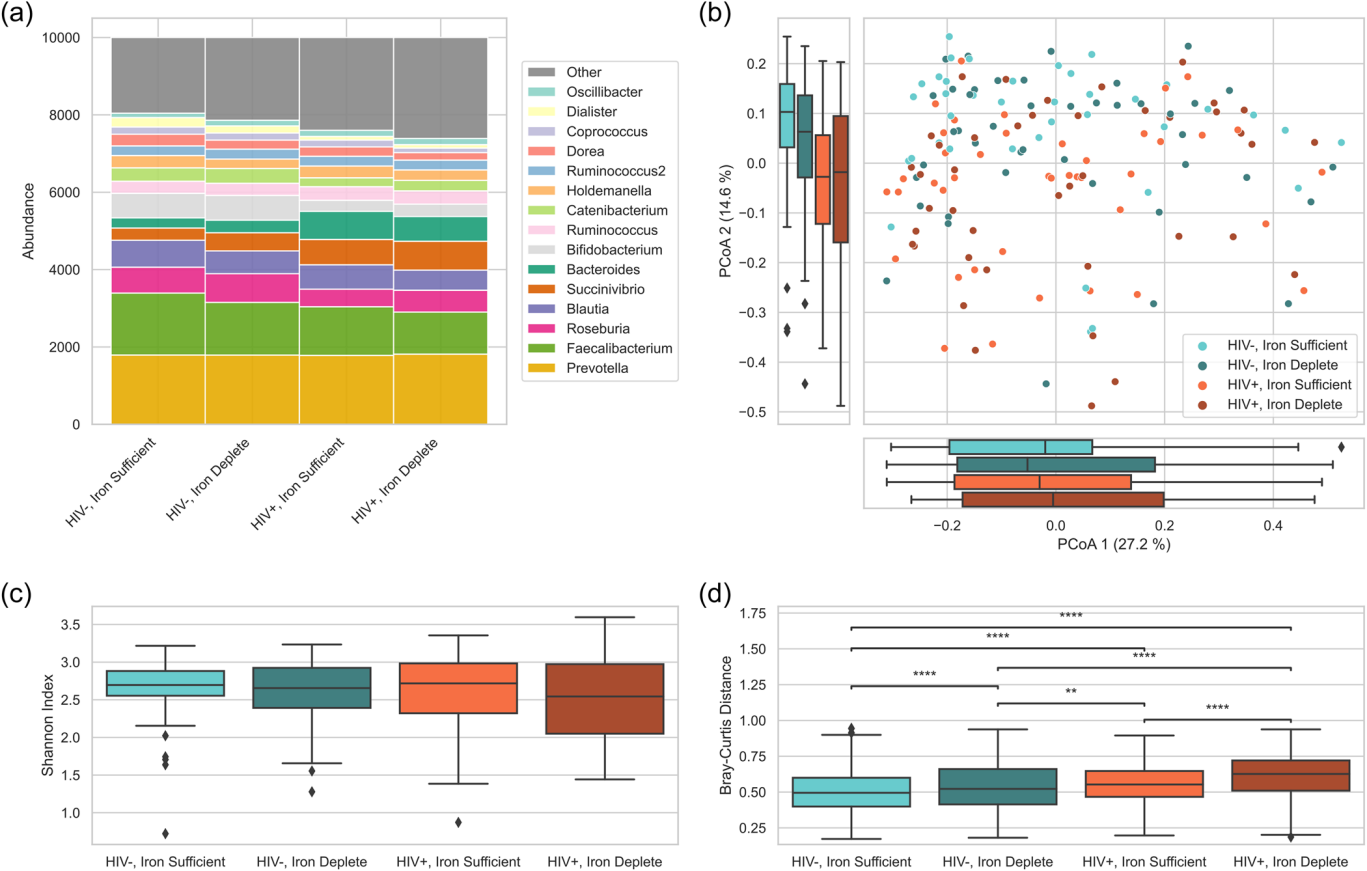
Relative abundance and diversity comparisons in virally suppressed HIV+ and HIV− children with sufficient iron stores (inflammation‐unadjusted plasma ferritin >40 µg/L) and insufficient iron stores (inflammation‐unadjusted plasma ferritin ≤40 µg/L). All children with sufficient iron stores were also nonanaemic (haemoglobin ≥115 g/L). (a) Relative abundance of faecal bacterial taxa at the genus level by HIV and iron status. Genera with low prevalence (detected in less than 20% of the samples) were excluded from the analysis. (b) Principal coordinate analysis of inter‐individual differences by Bray–Curtis dissimilarity. (c) Alpha diversity using the Shannon index in different groups; no significant difference was observed (Kruskal–Wallis). (d) Within‐group inter‐individual Bray–Curtis distance (β‐diversity); annotations above the box plots indicate significant difference between groups (Kruskal–Wallis followed by a post hoc Dunn's test with correction for multiple testing): **0.001 < *p* ≤ 0.01 and ****p ≤ 0.0001.

There was a high relative abundance of *Prevotella* in all four groups (Figure 1a). This was also observed during enterotyping, with 96.4% of all children presenting as the *Prevotella* enterotype and 3.6% as the *Bacteroides 2* enterotype. The PCoA (Figure 1b) revealed that along the first axis there was little variation between the four groups. However, along the second axis there was a clear downward shift for HIV+ samples, with samples from ID children spread out more. There were no significant differences in α‐diversity between the four groups (*p* = 0.99) (Figure 1c). β‐diversity (inter‐individual differences) based on mean Bray–Curtis distances was significantly higher in HIV+ than in HIV− children (*p* < 0.001) (Figure 1d). Because all HIV+ children were on ART, the effects of HIV status and ART cannot be uncoupled in this analysis.

The RDA, which unveils features in the metadata that drive variation in the microbiota, revealed that from a total of 35 variables (Supporting Information, Supplementary Table 1a and b), age was the only microbiota covariate (RDA *R* ^2^ = 0.016, *p* = 0.008). However, age showed a borderline difference (*p* = 0.06) by HIV status in this sample and may therefore capture some of the same variation as ART‐treated HIV. HIV status, when forced for first consideration, was a significant factor from the metadata (RDA *R* ^2^ = 0.009, *p* = 0.029), and age remained a significant factor (RDA *R* ^2^ = 0.013, *p* = 0.004).

Using probabilistic models with various degrees of complexity (see ‘Methods’ section), we examined the associations of HIV and iron status with the gut microbiota. Table 3 presents the differences in relative abundance explained by HIV status only (with no iron‐status effect). In contrast with traditional statistical methods, probabilistic methods do not provide *p*‐values but aim to generate a distribution of plausible values for a given parameter of interest. These distributions can be summarised by the smallest interval that contains 94% of those plausible values, the highest density interval (HDI) along with the mean and SD of those values.

**Table 3:** Genera with their relative abundance confidently linked with HIV status (without an effect of iron status) with their percentage change due to HIV status (mean and standard deviation from the probabilistic model) and the 94% highest density interval.

Genus	Relative abundance in children with no HIV or ID (per 10,000 reads)	Relative increase or decrease in abundance when HIV+ (%)	Highest density interval
More abundant in HIV+			
*Butyricimonas*	9.5	67	52–81
*Sutterella*	13.2	102	87–116
*Desulfomicrobium*	7.2	105	85–124
*Bacteroides*	309.7	112	109–115
*Alistipes*	42.7	130	121–139
*Clostridium_XlVb*	8.5	144	123–165
*Barnesiella*	14.3	150	134–167
*Desulfovibrio*	19.0	154	140–169
*Parabacteroides*	52.0	160	151–169
*Clostridium_XVIII*	16.2	180	163–197
*Bilophila*	4.5	202	168–237
*Phascolarctobacterium*	12.8	236	213–258
*Subdoligranulum*	1.5	253	189–324
*Odoribacter*	8.4	263	233–295
*Fusobacterium*	0.6	10,993	8108–14,058
Less abundant in HIV+			
*Turicibacter*	25.6	81	80–83
*Romboutsia*	37.3	71	69–73
*Intestinibacter*	18.2	63	60–67
*Clostridium_sensu_stricto*	58.0	57	55–59
*Terrisporobacter*	7.4	56	50–62
*Olsenella*	55.0	56	54–58
*Dialister*	208.2	55	54–56
*Bifidobacterium*	615.9	49	48–50
*Roseburia*	694.1	25	24–26
*Dorea*	264.2	17	16–19
*Faecalibacterium*	1437.8	16	16–17

**Figure 2:**
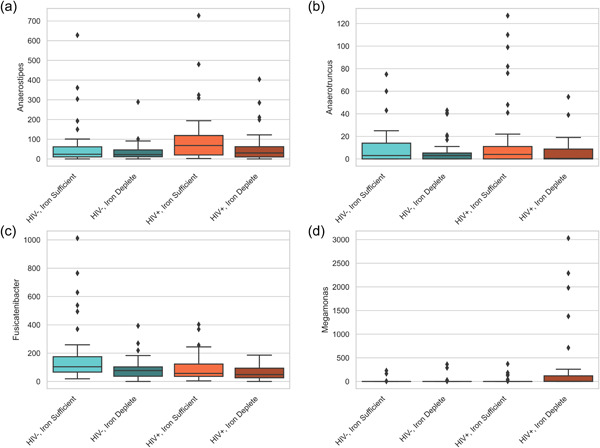
Example of four genera's relative abundance for which the probabilistic models confidently found a shift in abundance based on HIV and/or iron status. (a, b) *Anaerostipes* and *Anaerotruncus* are associated with iron status, (c) *Fusicatenibacter* is associated with both HIV and iron status independently and (d) *Megamonas* is the only genus where an HIV × iron status interaction effect was noted. The prevalence of *Megamonas* was higher in children with both HIV and ID versus HIV− and iron‐sufficient nonanaemic counterparts.

Models convincingly show that the relative abundances of *Anaerostipes* (Figure 2a) and *Anaerotruncus* (Figure 2b) shift by iron status but not by HIV status. The relative abundance of *Anaerostipes* and *Anaerotruncus* was 45% ± 1 (HDI 43–47) and 56% ± 2 (HDI 52–60) lower, respectively, in children with ID. *Fusicatenibacter* (Figure 2c) differed by HIV status and by iron status but with no interaction effect. *Fusicatenibacter* was 29% ± 1 (HDI 27–30) and 35% ± 1 (HDI 34–37) lower in children with HIV and in children with ID, respectively. There was an HIV × iron status interaction effect for *Megamonas* (Figure 2d). Given the low prevalence of *Megamonas*, an alternative model was tested. This model assumes a constant abundance across the different groups; however, the prevalence of people carrying *Megamonas* can differ between groups. This was a better fit for our data than the previous model, and although we cannot confidently say that *Megamonas* prevalence is affected by HIV or iron status alone, the prevalence was 42% ± 9 (HDI 25%–59%) higher in children with both HIV and ID than in HIV− and iron‐sufficient nonanaemic counterparts.

## DISCUSSION

Our aim was to better comprehend the associations of HIV and iron status with the gut health of 8‐ to 13‐year‐old South African children. We found that gut mucosal integrity and gut microbial α‐diversity measures did not significantly differ by HIV or iron status. ID was associated with higher gut inflammation, and both HIV and ID were associated with higher gut microbial β‐diversity. ART‐treated HIV and age explained the variance in gut microbiota composition across the four groups. In the HIV+ children, the relative abundance of 15 microbial genera was higher and that of 12 microbial genera was lower than in the HIV− children. In ID children, the relative abundance of *Anaerostipes*, *Anaerotruncus* and *Fusicatenibacter* was lower than in the iron‐sufficient nonanaemic children. There was an HIV × iron status interaction effect for *Megamonas*, and the prevalence‐based model confidently demonstrated that the prevalence of *Megamonas* was higher in children with both HIV and ID compared with HIV− and iron‐sufficient nonanaemic counterparts.

Encouragingly, we found no indication of gut mucosal barrier disruption according to plasma I‐FABP concentrations. Several studies have reported a loss of gut integrity in HIV+ individuals of varying age groups and support the chain of gastrointestinal events after HIV acquisition.^12^, ^35^, ^36^ However, I‐FABP is not consistently elevated in children and adults across studies. Our finding of no difference in I‐FABP by HIV status is consistent with previous studies in African adults^37^ and infants.^38^

Although increased faecal calprotectin was only suggestive of subclinical gut inflammation, the disparity between ID and iron‐sufficient children was significant, and ID, but not HIV, was associated with an increased faecal calprotectin. Previous studies have observed a positive association between faecal calprotectin and dysbiosis in the context of gastrointestinal diseases.^39^, ^40^ In our study, both HIV and ID contributed to dysbiosis (discussed further), and therefore, we expected an increase in faecal calprotectin in both HIV+ and ID children. Possibly, the mild gut inflammation observed was not a consequence of ID but rather a contributor to the depleted iron stores in ID children by impairing dietary iron absorption.

The gut microbiota diversity indices suggest that bacterial richness and evenness were similar between the HIV+ and HIV− children and independent of iron status. Previously, Abange et al. reported a lower Shannon index in Cameroonian HIV+ children and adolescents compared with HIV− counterparts,^41^ whereas this was not the case in a Zimbabwean study by Flygel et al.^42^ However, the latter group reported a decrease in α‐diversity in HIV+ children based on other diversity indices. Although these two studies included children and adolescents on ART, a substantial proportion were not virally suppressed. The participants either initiated treatment during study enrolment, or their time spent on ART was not sufficient to achieve viral suppression. Furthermore, one of these studies implemented a higher threshold for viral suppression (1000 HIV RNA copies/ml)^42^ than ours (50 HIV RNA copies/ml). Nowak et al. reported a positive correlation between gut microbial diversity and CD4+ T‐cell count, which is restored when viral load is suppressed.^43^ The comparable bacterial richness between HIV+ and HIV− children in our study may be the result of successful viral suppression, as observed in previous studies.^44^, ^45^

On the contrary, we detected a higher β‐diversity (dissimilarity between participants) in HIV+ than HIV− children, consistent with several paediatric and adult studies.^21^, ^41^, ^42^, ^43^ Within both the HIV+ and HIV− groups, ID children displayed significantly higher β‐diversity, suggesting that both HIV and ID can disrupt the microbiota and increase the variation within the composition. Thus, HIV and ID may additively contribute to dysbiosis.

Although *Prevotella* was previously associated with HIV,^46^, ^47^ our observation of a high relative abundance of *Prevotella* was not limited to HIV+ but observed in all four groups. Another recent study in young Capetonian children also reported a high relative abundance of *Prevotella*.^48^ This was not surprising as *Prevotella* can be enriched in individuals from nonindustrialised countries whose diets are high in fibre and low in protein.^49^

The RDA revealed that ART‐treated HIV and age were the only significant factors for explaining the variance in gut microbiota across the four groups. This was also confirmed by more significant differences in the relative abundances of bacterial genera across the four groups by HIV status than by iron status (27 versus 4 genera). HIV and ART are known modulators of the gut microbiota.^19^, ^50^ HIV‐related variation in gut microbial profiles differ by viraemia^51^, ^52^, ^53^ as well as by ART regimen.^50^, ^54^ Compared with recent HIV infection, an HIV‐specific gut microbiota signature depleted of *Akkermansia*, *Anaerovibrio*, *Bifidobacterium* and *Clostridium* appears to develop over time, becoming evident on long‐term ART.^55^ Of these genera and compared with HIV− counterparts, lower relative abundances of *Bifidobacteria* and *Clostridium* were noted in our sample of virally suppressed HIV+ children on ART. An interesting observation by HIV status was the steep increase in the relative abundance of *Fusobacterium* in the HIV+ children versus very low abundances in the HIV− children in our sample. *Fusobacterium* is a pathogenic bacterium with virulence factors that could trigger gut inflammation and disease.^56^ An increase in the relative abundance of *Fusobacterium* was previously associated with suboptimal immune recovery and functioning despite ART.^57^

In vitro and animal studies have highlighted the adverse effects of low colonic iron availability on butyrate‐producing bacteria and short‐chain fatty acid metabolism.^8^, ^9^ In our sample, the relative abundance of both *Anaerostipes* and *Anaerotruncus* was lower in ID children compared with iron‐sufficient nonanaemic counterparts. *Anaerostipes* and *Anaerotruncus* are butyrate‐producing probiotic bacteria strongly and positively correlated with Hb and serum ferritin in rats.^58^ The short‐chain fatty acid butyrate has anti‐inflammatory effects and is beneficial to intestinal health.^59^ Similar to our findings, a previous study among children with inflammatory bowel disease reported a lower abundance of butyrate‐producing microbiota when faecal calprotectin was elevated.^39^ Furthermore, our observation of a lower abundance of *Fusicatenibacter* in HIV+ as well as in ID children may also relate to gut inflammation, as a decrease in *Fusicatenibacter* has been associated with ulcerative colitis^60^ and Crohn's diseases,^61^ both chronic inflammatory diseases of the gut.

An HIV × iron status interaction effect was observed only for the genus *Megamonas*, with a higher prevalence of *Megamonas* in children with both HIV and ID than without HIV and ID. Although an increase in the relative abundance of *Megamonas* in ART‐treated HIV+ adults has been reported,^62^ literature associating *Megamonas* with ID is scarce. A study in women with gestational anaemia reported gut microbial enrichment of *Megamonas*.^63^ In HIV+ adults either on ART or ART naive, *Megamonas* was correlated significantly with interleukin‐6, a systemic inflammatory cytokine.^64^ In our study, HIV was significantly associated with increases in both CRP and AGP concentrations. Although the levels of inflammation were low, if considered together with the iron status–associated increase in gut inflammation, and HIV and iron status–associated changes in gut microbiota composition, our findings support an interplay between HIV, iron status, gut health and systemic inflammation.

Our study had several strengths. We investigated the relationship of two factors, HIV and iron status, with gut health. By using probabilistic models adapted specifically for this study design, interpretable results for the specific research questions could be obtained. Furthermore, the partially pooled models allow maximum information to be used from each participant's sample, reducing uncertainty of the results. A limitation of this study is that it was observational, and therefore, conclusions of causality or temporality cannot be drawn.

In conclusion, in 8‐ to 13‐year‐old virally suppressed HIV+ and HIV− children with or without ID, ID was associated with increased gut inflammation and changes in the relative abundance of specific microbiota. Moreover, in HIV+ children, ID had a cumulative effect that further shifted the gut microbiota to an unfavourable composition. Preventing ID in HIV+ children may benefit gut health, and supporting optimal iron status with context‐appropriate interventions should be prioritised.

## AUTHOR CONTRIBUTIONS

Charlene Goosen designed the study and conducted the research. Charlene Goosen, Kashish Mallick and Jeannine Baumgartner analysed the data (other than the microbiota data). Sebastian Proost performed the microbiota analysis and visualisation. Raul Y. Tito processed 16S sequencing data. Charlene Goosen and Kashish Mallick prepared the original draft of the paper. Shaun L. Barnabas, Mark F. Cotton and Michael B. Zimmermann provided study resources. Jeroen Raes supervised the microbiota analysis and provided study resources. Renée Blaauw supervised the research study and provided study resources. All authors reviewed the paper and read and approved the final manuscript.

## CONFLICT OF INTEREST STATEMENT

The authors declare no conflict of interest.

### PEER REVIEW

The peer review history for this article is available at https://www.webofscience.com/api/gateway/wos/peer-review/10.1111/jhn.13171.

## ETHICAL APPROVAL

The study was approved by the health research ethics committees of ETH Zurich (EK 2018‐N‐40) and Stellenbosch University (M18/05/017 and S18/06/136).

## TRANSPARENCY DECLARATION

The lead authors affirm that this manuscript is an honest, accurate and transparent account of the study being reported. The reporting of this work is compliant with STROBE guidelines. The lead authors affirm that no important aspects of the study have been omitted and that any discrepancies from the study as planned have been explained. The study cohort was from a trial registered at clinicaltrials.gov as NCT03572010.

## Supporting information

Supporting information.

## Data Availability

The data that support the findings of this study are available from the corresponding author upon reasonable request.
