# Supplementary material for: Associations of HIV and iron status with gut microbiota composition, gut inflammation and gut integrity in South African school‐age children: a two‐way factorial case–control study
Source: J Hum Nutr Diet. 2023 Apr 16;36(3):819–32. doi: 10.1111/jhn.13171 (PMC10946596; doi:10.1111/jhn.13171)
Supplement: Supplementary file 1 — Supporting information. [file JHN-36-819-s001.docx]

**SUPPLEMENTARY DATA**

**Supplementary Tables 1a and 1b** provide the full output of the redundancy analysis (RDA) with 35 features from the metadata, including non-significant results. The standard RDA, where the order of features is based on their non-redundant effect size, is shown in Supplementary Table 1a. As age and HIV status were associated (borderline significance) in this dataset, and HIV has a known effect on the microbiome, the analysis was repeated forcing HIV status to be included first. Results from the RDA with forced order are included in Supplementary Table 1b.

**Supplementary Table 1a.** Full RDA output

|  | **CAP_**  **F** | **CAP_**  **r2** | **CAP_**  **r2adj** | **CAP_**  **N** | **CAP_**  **p.value** | **CAP_**  **FDR** | **RDA**  **cumul_R2.adj** | **RDA**  **cumul_Df** | **RDA**  **cumul_AIC** | **RDA**  **cumul_F** | **RDA**  **cumul_Pr.F** | **Significant** |
| --- | --- | --- | --- | --- | --- | --- | --- | --- | --- | --- | --- | --- |
| Age | 2,908 | 0,022 | 0,016 | 158 | 0,003 | 0,112 | 0,016 | 1 | 547,486 | 2,908 | 0,008 | 1 |
| HIV status | 2,028 | 0,015 | 0,009 | 158 | 0,027 | 0,394 | 0,016 |  |  |  |  | 0 |
| Delivery mode | 1,745 | 0,013 | 0,007 | 158 | 0,058 | 0,394 | 0,016 |  |  |  |  | 0 |
| Non-haem iron intake | 1,633 | 0,012 | 0,006 | 158 | 0,077 | 0,394 | 0,016 |  |  |  |  | 0 |
| Animal protein intake | 1,603 | 0,012 | 0,006 | 158 | 0,089 | 0,394 | 0,016 |  |  |  |  | 0 |
| Height | 1,534 | 0,012 | 0,005 | 158 | 0,105 | 0,394 | 0,016 |  |  |  |  | 0 |
| Muscle protein intake | 1,476 | 0,011 | 0,005 | 158 | 0,117 | 0,394 | 0,016 |  |  |  |  | 0 |
| Iron status | 1,459 | 0,011 | 0,005 | 158 | 0,122 | 0,394 | 0,016 |  |  |  |  | 0 |
| Total protein intake | 1,430 | 0,011 | 0,005 | 158 | 0,132 | 0,394 | 0,016 |  |  |  |  | 0 |
| Calcium intake | 1,401 | 0,011 | 0,004 | 158 | 0,137 | 0,394 | 0,016 |  |  |  |  | 0 |
| Dietary fibre intake | 1,386 | 0,011 | 0,004 | 158 | 0,144 | 0,394 | 0,016 |  |  |  |  | 0 |
| Vitamin B12 intake | 1,359 | 0,010 | 0,004 | 158 | 0,150 | 0,394 | 0,016 |  |  |  |  | 0 |
| Weight | 1,358 | 0,010 | 0,004 | 158 | 0,153 | 0,394 | 0,016 |  |  |  |  | 0 |
| Thiamine intake | 1,335 | 0,010 | 0,004 | 158 | 0,172 | 0,394 | 0,016 |  |  |  |  | 0 |
| Height-for-age Z-score | 1,316 | 0,010 | 0,004 | 158 | 0,180 | 0,394 | 0,016 |  |  |  |  | 0 |
| Zn intake | 1,314 | 0,010 | 0,004 | 158 | 0,178 | 0,394 | 0,016 |  |  |  |  | 0 |
| Niacin intake | 1,291 | 0,010 | 0,003 | 158 | 0,197 | 0,403 | 0,016 |  |  |  |  | 0 |
| Folate intake | 1,254 | 0,010 | 0,003 | 158 | 0,209 | 0,403 | 0,016 |  |  |  |  | 0 |
| Plant protein intake | 1,239 | 0,009 | 0,003 | 158 | 0,219 | 0,403 | 0,016 |  |  |  |  | 0 |

|  | **CAP_**  **F** | **CAP_**  **r2** | **CAP_**  **r2adj** | **CAP_**  **N** | **CAP_**  **p.value** | **CAP_**  **FDR** | **RDA**  **cumul_R2.adj** | **RDA**  **cumul_Df** | **RDA**  **cumul_AIC** | **RDA**  **cumul_F** | **RDA**  **cumul_Pr.F** | **Significant** |
| --- | --- | --- | --- | --- | --- | --- | --- | --- | --- | --- | --- | --- |
| Iron intake | 1,215 | 0,009 | 0,003 | 158 | 0,233 | 0,408 | 0,016 |  |  |  |  | 0 |
| Haem iron intake | 1,180 | 0,009 | 0,003 | 158 | 0,256 | 0,426 | 0,016 |  |  |  |  | 0 |
| Bristol stool score | 1,143 | 0,009 | 0,002 | 158 | 0,286 | 0,455 | 0,016 |  |  |  |  | 0 |
| Zinc intake from fortified foods | 1,094 | 0,008 | 0,002 | 158 | 0,326 | 0,495 | 0,016 |  |  |  |  | 0 |
| Riboflavin intake | 1,025 | 0,008 | 0,001 | 158 | 0,383 | 0,558 | 0,016 |  |  |  |  | 0 |
| Vitamin B6 intake | 1,005 | 0,008 | 0,001 | 158 | 0,402 | 0,562 | 0,016 |  |  |  |  | 0 |
| pH | 0,984 | 0,008 | 0,001 | 158 | 0,430 | 0,577 | 0,016 |  |  |  |  | 0 |
| Calprotectin | 0,961 | 0,007 | 0,001 | 158 | 0,445 | 0,577 | 0,016 |  |  |  |  | 0 |
| Iron intake from fortified foods | 0,897 | 0,007 | 0,000 | 158 | 0,524 | 0,656 | 0,016 |  |  |  |  | 0 |
| Vitamin A intake | 0,847 | 0,006 | 0,000 | 158 | 0,576 | 0,695 | 0,016 |  |  |  |  | 0 |
| Phytate intake | 0,818 | 0,006 | 0,000 | 158 | 0,616 | 0,719 | 0,016 |  |  |  |  | 0 |
| Sex | 0,787 | 0,006 | 0,000 | 158 | 0,651 | 0,735 | 0,016 |  |  |  |  | 0 |
| Vitamin C intake | 0,688 | 0,005 | -0,001 | 158 | 0,782 | 0,856 | 0,016 |  |  |  |  | 0 |
| Intestinal fatty acid-binding protein | 0,647 | 0,005 | -0,001 | 158 | 0,824 | 0,868 | 0,016 |  |  |  |  | 0 |
| C-reactive protein | 0,629 | 0,005 | -0,002 | 158 | 0,844 | 0,868 | 0,016 |  |  |  |  | 0 |
| Alpha-1-acid glycoprotein | 0,595 | 0,005 | -0,002 | 158 | 0,875 | 0,875 | 0,016 |  |  |  |  | 0 |

**Supplementary Table 1b.** Full RDA output with HIV status forced to be considered first

|  | **CAP_**  **F** | **CAP_**  **r2** | **CAP_**  **r2adj** | **CAP_**  **N** | **CAP_**  **p.value** | **CAP_**  **FDR** | **RDA**  **cumul_R2.adj** | **RDA**  **cumul_Df** | **RDA**  **cumul_AIC** | **RDA**  **cumul_F** | **RDA**  **cumul_Pr.F** | **Significant** |
| --- | --- | --- | --- | --- | --- | --- | --- | --- | --- | --- | --- | --- |
| HIV status | 2,028 | 0,015 | 0,009 | 158 | 0,026 | 0,385 | 0,009 | 1 | 548,364 | 2,028 | 0,029 | 1 |
| Age | 2,908 | 0,022 | 0,016 | 158 | 0,003 | 0,102 | 0,022 | 1 | 547,749 | 2,587 | 0,004 | 1 |
| Delivery mode | 1,745 | 0,013 | 0,007 | 158 | 0,057 | 0,385 | 0,022 |  |  |  |  | 0 |
| Non-haem iron intake | 1,633 | 0,012 | 0,006 | 158 | 0,081 | 0,385 | 0,022 |  |  |  |  | 0 |
| Animal protein intake | 1,603 | 0,012 | 0,006 | 158 | 0,087 | 0,385 | 0,022 |  |  |  |  | 0 |
| Height | 1,534 | 0,012 | 0,005 | 158 | 0,105 | 0,385 | 0,022 |  |  |  |  | 0 |
| Muscle protein intake | 1,476 | 0,011 | 0,005 | 158 | 0,120 | 0,385 | 0,022 |  |  |  |  | 0 |
| Iron status | 1,459 | 0,011 | 0,005 | 158 | 0,125 | 0,385 | 0,022 |  |  |  |  | 0 |
|  | **CAP_**  **F** | **CAP_**  **r2** | **CAP_**  **r2adj** | **CAP_**  **N** | **CAP_**  **p.value** | **CAP_**  **FDR** | **RDA**  **cumul_R2.adj** | **RDA**  **cumul_Df** | **RDA**  **cumul_AIC** | **RDA**  **cumul_F** | **RDA**  **cumul_Pr.F** | **Significant** |
| Total protein intake | 1,430 | 0,011 | 0,005 | 158 | 0,133 | 0,385 | 0,022 |  |  |  |  | 0 |
| Calcium intake | 1,401 | 0,011 | 0,004 | 158 | 0,148 | 0,385 | 0,022 |  |  |  |  | 0 |
| Dietary fibre intake | 1,386 | 0,011 | 0,004 | 158 | 0,144 | 0,385 | 0,022 |  |  |  |  | 0 |
| Vitamin B12 intake | 1,359 | 0,010 | 0,004 | 158 | 0,157 | 0,385 | 0,022 |  |  |  |  | 0 |
| Weight | 1,358 | 0,010 | 0,004 | 158 | 0,159 | 0,385 | 0,022 |  |  |  |  | 0 |
| Thiamine intake | 1,335 | 0,010 | 0,004 | 158 | 0,171 | 0,385 | 0,022 |  |  |  |  | 0 |
| Height-for-age Z-score | 1,316 | 0,010 | 0,004 | 158 | 0,173 | 0,385 | 0,022 |  |  |  |  | 0 |
| Zn intake | 1,314 | 0,010 | 0,004 | 158 | 0,176 | 0,385 | 0,022 |  |  |  |  | 0 |
| Niacin intake | 1,291 | 0,010 | 0,003 | 158 | 0,190 | 0,391 | 0,022 |  |  |  |  | 0 |
| Folate intake | 1,254 | 0,010 | 0,003 | 158 | 0,204 | 0,396 | 0,022 |  |  |  |  | 0 |
| Plant protein intake | 1,239 | 0,009 | 0,003 | 158 | 0,215 | 0,396 | 0,022 |  |  |  |  | 0 |
| Iron intake | 1,215 | 0,009 | 0,003 | 158 | 0,232 | 0,406 | 0,022 |  |  |  |  | 0 |
| Haem iron intake | 1,180 | 0,009 | 0,003 | 158 | 0,252 | 0,419 | 0,022 |  |  |  |  | 0 |
| Bristol stool score | 1,143 | 0,009 | 0,002 | 158 | 0,277 | 0,441 | 0,022 |  |  |  |  | 0 |
| Zinc intake from fortified foods | 1,094 | 0,008 | 0,002 | 158 | 0,328 | 0,499 | 0,022 |  |  |  |  | 0 |
| Riboflavin intake | 1,025 | 0,008 | 0,001 | 158 | 0,385 | 0,562 | 0,022 |  |  |  |  | 0 |
| Vitamin B6 intake | 1,005 | 0,008 | 0,001 | 158 | 0,410 | 0,567 | 0,022 |  |  |  |  | 0 |
| pH | 0,984 | 0,008 | 0,001 | 158 | 0,422 | 0,567 | 0,022 |  |  |  |  | 0 |
| Calprotectin | 0,961 | 0,007 | 0,001 | 158 | 0,445 | 0,577 | 0,022 |  |  |  |  | 0 |
| Iron intake from fortified foods | 0,897 | 0,007 | 0,000 | 158 | 0,513 | 0,642 | 0,022 |  |  |  |  | 0 |
| Vitamin A intake | 0,847 | 0,006 | 0,000 | 158 | 0,573 | 0,692 | 0,022 |  |  |  |  | 0 |
| Phytate intake | 0,818 | 0,006 | 0,000 | 158 | 0,624 | 0,728 | 0,022 |  |  |  |  | 0 |
| Sex | 0,787 | 0,006 | 0,000 | 158 | 0,656 | 0,741 | 0,022 |  |  |  |  | 0 |
| Vitamin C intake | 0,688 | 0,005 | -0,001 | 158 | 0,779 | 0,852 | 0,022 |  |  |  |  | 0 |
| Intestinal fatty acid-binding protein | 0,647 | 0,005 | -0,001 | 158 | 0,828 | 0,865 | 0,022 |  |  |  |  | 0 |
| C-reactive protein | 0,629 | 0,005 | -0,002 | 158 | 0,840 | 0,865 | 0,022 |  |  |  |  | 0 |
| Alpha-1-acid glycoprotein | 0,595 | 0,005 | -0,002 | 158 | 0,881 | 0,881 | 0,022 |  |  |  |  | 0 |
